# Intergrading reef communities across discrete seaweed habitats in a temperate–tropical transition zone: Lessons for species reshuffling in a warming ocean

**DOI:** 10.1002/ece3.8538

**Published:** 2022-01-24

**Authors:** Yannick Mulders, Karen Filbee‐Dexter, Sahira Bell, Nestor E. Bosch, Albert Pessarrodona, Defne Sahin, Sofie Vranken, Salvador Zarco‐Perello, Thomas Wernberg

**Affiliations:** ^1^ UWA Oceans Institute and School of Biological Sciences Perth WA Australia; ^2^ Institute of Marine Research Bergen Norway; ^3^ Department of Science and Environment Roskilde University Roskilde Denmark

**Keywords:** fish ecology, invertebrate ecology, ocean warming, seaweed ecology, species distribution, subtidal reefs, temperate‐to‐tropical transition zone

## Abstract

Temperate reefs are increasingly affected by the direct and indirect effects of climate change. At many of their warm range edges, cool‐water kelps are decreasing, while seaweeds with warm‐water affinities are increasing. These habitat‐forming species provide different ecological functions, and shifts to warm‐affinity seaweeds are expected to modify the structure of associated communities. Predicting the nature of such shifts at the ecosystem level is, however, challenging, as they often occur gradually over large geographical areas. Here, we take advantage of a climatic transition zone, where cool‐affinity (kelp) and warm‐affinity (*Sargassum*) seaweed forests occur adjacently under similar environmental conditions, to test whether these seaweed habitats support different associated seaweed, invertebrate, coral, and fish assemblages. We found clear differences in associated seaweed assemblages between habitats characterized by kelp and *Sargassum* abundance, with kelp having higher biomass and seaweed diversity and more cool‐affinity species than *Sargassum* habitats. The multivariate invertebrate and fish assemblages were not different between habitats, despite a higher diversity of fish species in the *Sargassum* habitat. No pattern in temperature affinity of the invertebrate or fish assemblages in each habitat was found, and few fish species were exclusive to one habitat or the other. These findings suggest that, as ocean warming continues to replace kelps with *Sargassum*, the abundance and diversity of associated seaweeds could decrease, whereas fish could increase. Nevertheless, the more tropicalized seaweed habitats may provide a degree of functional redundancy to associated fauna in temperate seaweed habitats.

## Graphical abstract

We investigated seaweed, coral, mobile invertebrate, and fish communities in quintessentially cool and warm seaweed habitats in a transition zone. There was a discrete separation between the temperature affinity of sessile benthic organisms. This separation was not reflected in the relative abundance of fish and mobile invertebrates in each habitat.

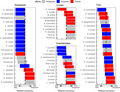

## 1 INTRODUCTION

Changing species distributions are some of the most pervasive effects of global warming on natural ecosystems of the world (Pecl et al., 2017). The geographic distribution of many groups of marine species has been altered through changes in habitat suitability, dispersal trajectories, and mortality rates (Beaugrand et al., 2008; Chen et al., 2011; Perry et al., 2005; Poloczanska et al., 2013; Wernberg, Bennett, et al., 2016), often with substantial socio‐economic consequences (Free et al., 2019; Smale et al., 2019; Thiault et al., 2019). These changes have been documented extensively in coastal locations, and their prevalence is predicted to intensify as global warming continues (Assis et al., 2018; Cheung et al., 2009; Martínez et al., 2018; Molinos et al., 2015; Wilson et al., 2019).

The reorganization of marine biodiversity across the globe is often characterized by expansions, contractions, or range shifts toward higher latitudes (Blowes et al., 2019; Lonhart et al., 2019; Vergés, Steinberg, et al., 2014). Among other environmental drivers (*e*.*g*., storms, acidification, or sedimentation), increasing temperatures drive species with affinities to warmer temperature (*e*.*g*., tropical species) into temperate ecosystems, a phenomenon commonly known as “tropicalization” (Vergés, Tomas, et al., 2014). The range shifts and persistence of species are dependent on their distinctive thermal tolerances, different acclimatization and adaptation capacities, dispersal abilities, and biological interactions, such as the presence of adequate habitat and food resources, competition, and predation (Gilman et al., 2010; Van der Putten et al., 2010). Range shifts of one species can also decouple ecological interactions if other species responses are incompatible within the range (Schweiger et al., 2008), adding further complexity to ecosystem‐level responses to ocean warming. Therefore, the mixing and rearranging of species with differing temperature affinity can lead to novel community compositions (Urban et al., 2012; Vergés, Steinberg, et al., 2014; Williams et al., 2007).

As progressing tropicalization forces increasing convergence of temperate and tropical species, the classic notion of discreteness between temperate and tropical ecosystems is blurring (Pinsky et al., 2020; Stuart‐Smith et al., 2017). Worldwide, the pole‐ward shift of species has been reported for many taxa belonging to different trophic levels and functional groups (Jones & Cheung, 2015). These include sessile and benthic species such as seaweeds and seagrasses (Hyndes et al., 2016; Wernberg et al., 2011), echinoderms, mollusks (Mulders & Wernberg, 2020), hermatypic corals (Price et al., 2019), and highly mobile fauna such as fish (Hastings et al., 2020; Vergés, Tomas, et al., 2014). However, changes in the composition of foundation species will have the greatest consequences for the functioning of the ecosystems (Vergés et al., 2019). In temperate reefs, canopy‐forming seaweed of the order laminariales (kelp) is a dominant foundation species that control the community structure by providing shelter and food to many species, as well as modifying the environmental conditions (through shading, current dampening, particle entrainment) to facilitate some species over others (Cavanaugh et al., 2019; Wernberg et al., 2019). Thus, changes in the composition of such species can have strong effects across multiple trophic levels (Norderhaug et al., 2020; Teagle et al., 2017).

Kelp forests are in decline in many regions of the world, and this is forecasted to continue in the future due to the direct and indirect effects of climate change (Martínez et al., 2018; Wernberg et al., 2019). This has led to impoverished ecosystem states dominated by turf algae or sea urchin barrens in some regions (Filbee‐Dexter & Wernberg, 2018; Ling et al., 2010; Rogers‐Bennett & Catton, 2019); however, in some places, the primary foundation species in kelp forests have been replaced with alternative foundation species, such as invasive seaweeds (Filbee‐Dexter et al., 2016; Thomsen et al., 2019), or seaweeds with a more tropical affinity, such as *Sargassum* species (e.g., Engelen et al., 2008; Serisawa et al., 2004), which is predicted to be one of the key future scenarios for kelp forests more broadly (Vergés et al., 2019). The outcomes of this replacement are likely to have important consequences for the services that these ecosystems provide and thus is of paramount importance to understand the possible consequences of the rearrangement of their communities in the future (Beas‐Luna et al., 2020; Pessarrodona et al., 2019; Vergés et al., 2019).

Due to warming and periodically increasing flow of the southwards‐flowing Leeuwin Current over the past couple of decades, Western Australia (WA) has become one of the tropicalization hot spots of the world. As a result, tropical fauna has infiltrated into higher latitudes (Hyndes et al., 2016; Richards et al., 2016; Wernberg, Bennett, et al., 2016; Zarco‐Perello et al., 2017), while kelp forests are experiencing range contractions, which is happening concurrently with increases in the abundance of *Sargassum* species (Wernberg et al., 2016). This suggests that a shift from kelp to *Sargassum* dominance—and possibly eventually even coral dominance—could be a possible outcome of future warming in this region (Martínez et al., 2018; Tuckett et al., 2017; Vergés et al., 2019).

Assessing the effects of the substitution of habitat providers due to climate change in temperate reefs is difficult, particularly in natural settings, because these changes occur gradually over long temporal scales. However, changes over time in a larger geographical area are expected to be similar to changes over space within constrained climatic transition zones (Agostini et al., 2018; Vergés, Steinberg, et al., 2014; Wernberg et al., 2012). By utilizing this “space‐for‐time substitution” approach, existing transition zones between temperate and tropical boundaries are particularly useful to study the effects of global warming, providing unique insights into possible trajectories of ecosystems in these rapidly shifting regions. In particular, these climatic transition zones, where different habitat configurations co‐exist in adjacent areas under similar environmental conditions and propagule supply, provide an opportunity to disentangle the influence of changing foundation species and shifting species distributions on the trajectory of communities.

This study focused on the Houtman Abrolhos archipelago, which is located ~80 km off the coast of Geraldton in the midwest of Western Australia. The archipelago is positioned within the main flow of the Leeuwin Current, which runs south from tropical to temperate latitudes, carrying warm oligotrophic waters and many tropical species (Hutchins & Pearce, 1994; Phillips & Huisman, 2009). As a result, the ecosystems here are a mosaic of habitats of temperate kelp forests and tropical coral gardens and *Sargassum* meadows, making it an ideal location to investigate the possible consequences at ecosystem level of a shift in habitat structure. By conducting a wider ecosystem functioning analysis, we tested whether kelp (cool affinity) and *Sargassum* (warm affinity)‐dominated reefs occurring in the same general area support different associated seaweed, invertebrate, coral, and fish assemblages. We also assessed the associated climatic affinity of the communities found in these two habitats to gain insights into the potential downstream effects of a temperate to tropical shift, and the potential for redundancy in functions provided by different foundation species.

## 2 MATERIALS AND METHODS

### 2.1 Site selection

**FIGURE 1:**
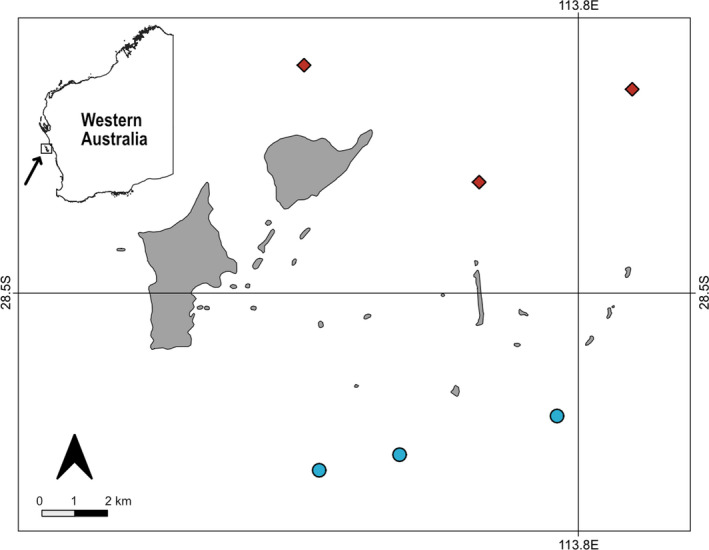
Sampling sites at the Wallabi Group of the Houtman Abrolhos archipelago. Sargassum habitats are indicated with a red diamond, and Kelp habitats are indicated with a blue circle

All fieldwork was conducted in October 2019 in the Australasian spring around the Wallabi Island Group of the Houtman Abrolhos archipelago (28°43′S; 113°47′E), in midwestern Western Australia (Figure 1). Potential sites were identified on navigational charts where the reef was between 8 and 12 m of depth and separated by a minimum distance of 2 km from any other site. Six sites were selected based on visual confirmation of desired habitat type (three kelp and three *Sargassum* habitats; hereafter referred to as “Kelp habitat” and “*Sargassum* habitat”). All study sites were located on gradually sloping limestone reefs. While wave exposure data on small spatial scales are not available for this region, the Kelp habitats are more directly exposed to the predominant oceanic swell coming from the southwest. Due to the remoteness of the area, there are no long‐term data available on the benthic seaweed community composition at the site level.

### 2.2 Sample collection

At all sites, taxonomic groups were sampled sequentially. First, we surveyed fish communities to minimize the effect of diver presence on fish counts, followed by seaweeds, mobile invertebrates, and corals. Fish were sampled using stereo‐DOV (Diver Operated Video) surveys. Eight replicate transects of 25 × 5 m (125 m^2^) were sampled at each site. Surveys were conducted in a minimum of 7‐m visibility with 10‐m separation between each replicate. Transects were conducted by a team of two SCUBA divers, whereby one swam at constant speed along each transect with the stereo‐DOV, while the other measured distance with a tape measure. This ensured that the effects of SCUBA diver presence were minimized, with only one diver present with the cameras (Watson & Harvey, 2007). The stereo‐DOV system consisted of two GoPro Hero 4 video cameras in underwater housings, mounted 0.7 m apart on an aluminum frame, and converged at 8° to provide a standardized field of view (from 0.5 to 8 m). A complete description of stereo‐DOVs including an explanation of how they are configured and calibrated is described by Goetze et al. (2019). Videos were analyzed using the program EventMeasure (Stereo, www.seagis.com.au) for species identification, size (fork length). Biomass was then calculated using estimates based on length–weight relationships obtained from FishBase (Froese & Pauly, 2021). Settings were established to maintain the transect limits and to prevent fish more than 7 m from the camera from being included in the analysis. Fish that were not visible in both cameras were unable to be measured but remained included in the density data if the fish was within the transect boundaries. Vagrant schooling species were omitted from the analysis to minimize type I errors.

Seaweeds were sampled independently with six 0.25‐m^2^ quadrats haphazardly placed on the benthos. All seaweed specimens visible to the unaided eye were plucked (taking care to include the holdfast) from the substrate and collected in calico bags. Large seaweeds were sorted on the day of collection and identified to lowest taxonomic level possible. Smaller and harder‐to‐identify specimens were frozen and identified later in the laboratory. Fresh weight of each species was determined using a digital scale, after removing excess water.

Mobile invertebrates were sampled along five 5‐m transects at each site, haphazardly positioned on the reef flat and separated by at least 5 m. For each transect, 0.5 m of the benthos at both sides was examined for invertebrates larger than 10 mm. Encountered specimens were identified to the lowest taxonomic level possible and counted for abundance. Corals were sampled within five 1‐m^2^ quadrats haphazardly placed on the benthos. All coral colonies visible to the unaided eye were identified to lowest taxonomic level possible and counted for abundance.

### 2.3 Temperature affinities

All seaweed, invertebrate, coral, and fish species were classified into three temperature affinity categories: “warm affinity,” “cool affinity,” and “cosmopolitan.” Seaweed temperature affinities were determined by known thermal preferences and distribution range for the species (Herbarium, 1998; Huisman, 1997, 2019). Temperature affinities of invertebrate and coral species were determined by known thermal preference. If the temperature affinity was unknown, when the proportion of occurrences was over 25% at a lower latitude than 28.3°S (Atlas of Living Australia, 2020) species were considered of warm affinity, while species that occurred over 75% at a higher latitude than 28.3°S were considered cool. Species that did not show a fit into either affinity based on known preference or distribution were considered cosmopolitan. See Tables S2–S5 for the temperature affinities of species.

### 2.4 Environmental data

To compare environmental conditions between the two habitat types, satellite‐derived long‐term temperature and nutrient measurements were sourced from the Integrated Marine Observing System (IMOS) using the “SRS–SST–L3S–6 day–day and night time” single sensor for sea surface temperature (°C) and the “SRS–MODIS–01 day–chlorophyll‐a concentration (OC3 model)” sensor for chlorophyll levels (mg m^−3^) (IMOS, 2019). For each site, mean and minimum monthly sea surface temperature (SST) was calculated from available daily means from 1992 through 2019. Chlorophyll concentration of the surface water was used as a proxy for nutrient content (Russell et al., 2005). Mean chlorophyll levels were calculated from available daily concentrations from 2002 through 2019.

### 2.5 Statistical analyses

All statistical analyses were done in R_3.6.2_ (R Core Team, 2019). Differences in univariate assemblage attributes between habitats were analyzed by creating generalized linear mixed models (GLMMs) using the “glmer” function from “lme4” package (Bates et al., 2007). For the seaweed models, *E*. *radiata* and *Sargassum* spp. were included in the analysis in the response variables. All models used Habitat as fixed factor (2 levels: Kelp vs. *Sargassum*) and Site as random effect (3 per habitat), with quadrat/transect nested within Site (8 for fish, 6 for seaweed, and 5 for invertebrate per site). As the data were zero‐skewed, richness and abundance count models were fitted using a Poisson distribution and include a logarithmic link function. Biomass models were fitted using a Gamma distribution with an inverse link function. The appropriateness of the fitted models was checked by visually inspecting the residuals using the “ggResidpanel” package. Dissimilarities in community composition between habitats were visualized by principle coordinates analysis (PCO) and analyzed by analysis of similarities (ANOSIM), and similarity percentage (SIMPER) analysis, using the “vegan” package (Oksanen et al., 2010). Abundance data were log‐transformed, and the Bray–Curtis dissimilarity of each point was then determined. The first two dimensions were used to plot the PCO, and the ANOSIM performed to determine the similarity of the points between habitats. SIMPER analysis was used to determine which species accounted for the largest amount of dissimilarity between habitats. The top 20 species of fish and seaweeds identified in the SIMPER analysis were classified by thermal affinity. For invertebrates, the species that made up to 98% of the dissimilarity were used, as there were less species compared with seaweeds and fish. A Mann–Whitney *U* test was then performed on the relative abundance of species with warm and cool affinities, comparing the ranks of warm species to the ranks of cool species for each habitat. Probability densities of the fish size were generated using kernel density estimates (KDE) of the pooled samples for each habitat, using a bandwidth 18.11 mm which was determined using Silverman's rule of thumb (Silverman, 1998). Dissimilarities of the size distributions between habitats were then tested using a two‐sample Kolmogorov–Smirnov test.

## 3 RESULTS

**FIGURE 2:**
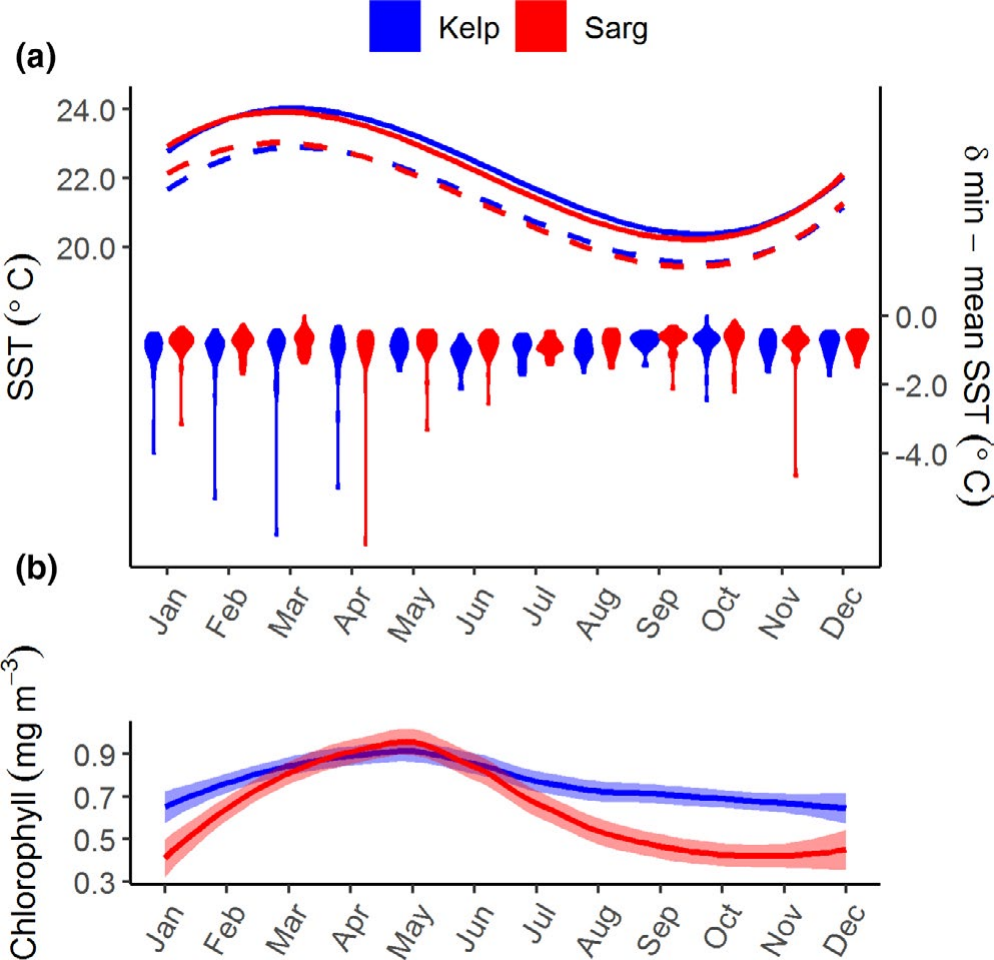
Seasonal trends in sea surface temperature and pelagic productivity at Houtman Abrolhos archipelago. (a) The average (solid line) and minimum (dashed line) monthly sea surface temperature (SST), and the difference between the average and minimum SST (violin plots; secondary *y*‐axis) between 1992 and 2019 on Kelp (blue) and Sargassum (red) habitats. (b) Differences in seasonal trends in average chlorophyll concentration between Kelp (blue) and Sargassum habitats

On decadal time scales, the average temperature profile for sites for each habitat was nearly identical for ecological purposes (Figure 2), although the Kelp habitat was slightly warmer between March and October (*F*_1,639_ = 4.956, *p* = .026). While minimum temperatures in the between habitats did not differ (*F*_1,639_ = 0.034, *p* = .854), the difference between the mean and minimum temperature was larger in the Kelp habitats (*F*_1,639_ = 5.475, *p* = .020). Chlorophyll content indicated both habitats were nutrient poor, although chlorophyll was higher at sites in the Kelp habitat in winter (*F*_1,301_ = 47.099, *p* < .001). Also, see Table S1 for additional details on statistical analysis of environmental data.

**FIGURE 3:**
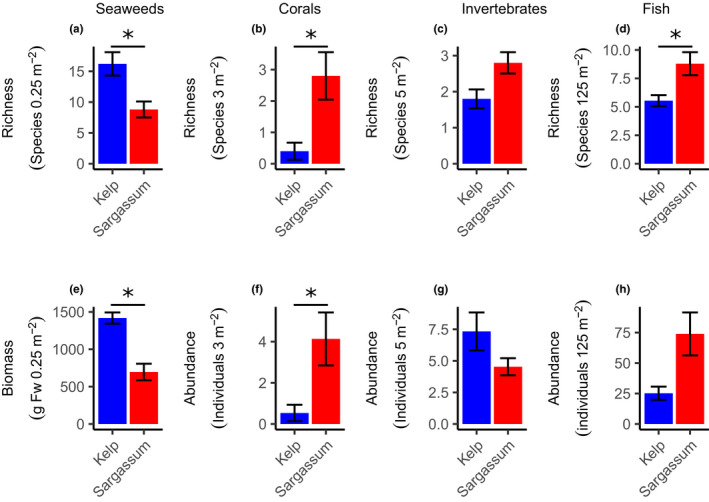
Mean species richness and abundance of seaweeds (a, e; including *E. radiata* and *Sargassum* spp., *n* = 18 per habitat), corals (b, f; *n* = 15), mobile invertebrates (c, g; *n* = 15), and fish (d, h; *n* = 24), in Kelp (blue) and Sargassum (red) habitats at the Houtman Abrolhos archipelago. Error bars indicate standard error, and asterisk indicates significant difference (GLMM, *p* < .05, see Table 1)

There was a clear difference between the Kelp and *Sargassum* habitats for the univariate measures of the seaweed and fish communities (Table 1). In the Kelp habitats, the seaweeds were more diverse (Figure 3a), had higher biomass (Figure 3d), and were characterized by a mixed assemblage of brown and red seaweeds such as *Callophycus oppositifolius*, *Hennedya crispa*, and *Pterocladia lucida*. In contrast, *Sargassum* habitats were largely dominated by a range of *Sargassum* species (92.4% of total biomass), featured a relatively impoverished seaweed understory and epiphytic assemblages (see Table S2 for details). For the invertebrate community, the difference between Kelp and *Sargassum* habitats was less pronounced. While the diversity (Figure 3b) was higher in the *Sargassum* habitats due to a diverse selection of gastropods, there was a higher density in the Kelp habitat (Figure 3e), due to the abundance of the urchin *Centrostephanus tenuispinus*. Fish were more diverse (Figure 3c) and abundant (Figure 3f) in the *Sargassum* habitats. In the Kelp habitat, there was a predominance of wrasses (Labridae), while in the *Sargassum* habitats, there was a more mixed community of wrasses, parrotfish (Scaridae), and damselfish (Pomacentridae). In total, 2521 individual fish from 51 species within 20 families were recorded across all sites. Of those, 45 and 26 species were found within 17 and 9 families in the *Sargassum* and Kelp habitat, respectively. Thus, only six species within three families were unique to the Kelp habitats.

**TABLE 1:** The GLMM outputs, testing for differences in univariate community attributes (richness, abundance, and biomass) between Kelp and Sargassum habitats as fixed factors. The four taxonomic groups considered: seaweed, corals, invertebrates, and fish

Model	Family	Link	DF (Residual DF)	Estimate	SE	*Z* value	Pr(>|z|)
Seaweed							
Richness	Poisson	Log	1 (32)	0.6811	0.1715	3.971	**<.001**
Biomass	Gamma	Inverse	1 (32)	0.0008	0.0002	4.817	**<.001**
Epiphyte	Gamma	Inverse	1 (32)	0.0248	0.0193	1.286	.199
Canopy	Gamma	Inverse	1 (32)	0.0004	0.0011	0.359	.720
Understorey	Gamma	Inverse	1 (32)	0.0476	0.0080	5.986	**<.001**
Invertebrates							
Richness	Poisson	Log	1 (27)	0.4418	0.2467	1.791	.073
Abundance	Poisson	Log	1 (27)	0.4429	0.3018	1.467	.142
Corals							
Richness	Poisson	Log	1 (27)	1.8636	0.7803	2.388	.**017**
Abundance	Poisson	Log	1 (27)	1.9636	0.9297	2.112	.**035**
Fish							
Richness	Poisson	Log	1 (45)	0.4396	0.2164	2.031	.**042**
Abundance	Poisson	Log	1 (45)	0.8551	0.4972	1.720	.086
Herbivore	Poisson	Log	1 (45)	1.1591	0.5820	1.992	.**046**
Invertivore	Poisson	Log	1 (45)	0.0145	0.3485	0.042	.967
Carnivore	Poisson	Log	1 (45)	0.1799	0.6595	0.273	.785
Planktivore	Poisson	Log	1 (45)	3.0582	2.1468	1.425	.154
Biomass	Gamma	Inverse	1 (44)	0.3338	0.0095	35.190	**<.001**
Herbivore	Gamma	Inverse	1 (44)	0.0009	0.0016	0.563	.573
Invertivore	Gamma	Inverse	1 (44)	0.0010	0.0003	3.255	.**001**
Carnivore	Gamma	Inverse	1 (44)	0.0001	0.0029	0.047	.962
Planktivore	Gamma	Inverse	1 (44)	0.0354	0.0331	1.069	.285

Bold values indicate statistically significant difference (*p* < .05).

**FIGURE 4:**
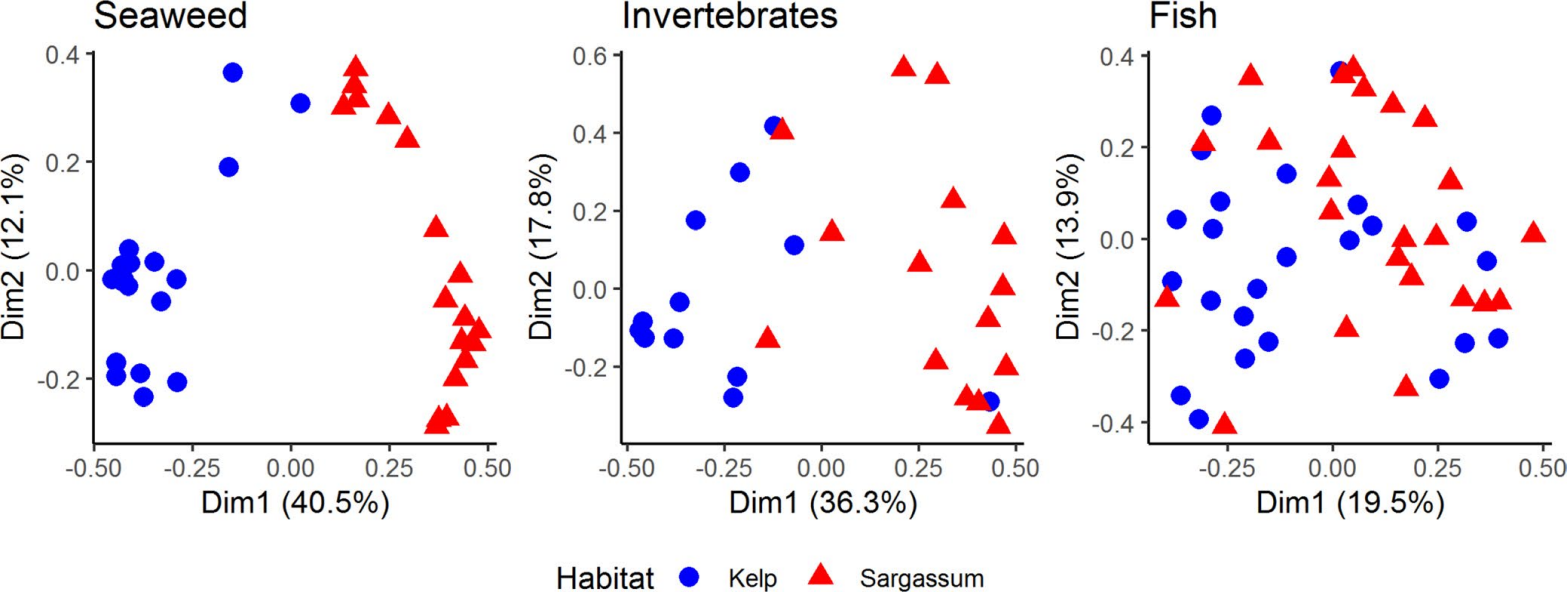
PCO of the Bray–Curtis dissimilarities for seaweed biomass (including *Sargassum* spp. and *Ecklonia radiata*, *n* = 18 per habitat), invertebrate abundance (*n* = 15), and fish abundance (*n* = 24) in the Abrolhos. Data were log‐transformed and grouped by Kelp (blue circles) and Sargassum (red triangles) habitats

**FIGURE 5:**
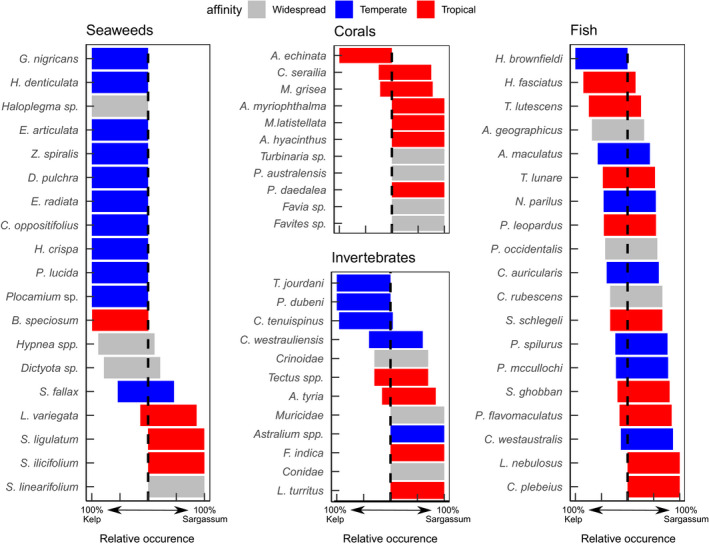
Relative occurrence between Kelp and Sargassum habitats of the most relevant species (from SIMPER analysis) of seaweeds, corals, mobile invertebrates, and fish in the Abrolhos. Warm temperature‐associated species are red, cool temperature‐associated species are blue, and cosmopolitan species without a clear temperature affinity are gray

Comparing the habitats, there was a clear separation between the seaweed communities (ANOSIM: *R* = .921, *p* = .001), a slight separation for the invertebrates (ANOSIM: *R* = .490, *p* = .001), and almost no separation in the fish communities (ANOSIM: *R* = .194, *p* = .001) (Figure 4). Of the 20 species of seaweeds that drove the majority of the dissimilarity between habitats, 80% were only found in one habitat type. For invertebrates, about one‐third of the species were found in both habitats. Finally, most fish were present in both habitats, with only four species restricted to a single habitat (Figure 5).

Seaweed species with cool temperature affinities were predominantly found in the Kelp habitats (Figure 5; Mann–Whitney *U*: *U* = 41.5, *p* = .026). Similarly, invertebrates with cool affinities were relatively more abundant in the Kelp habitats (Mann–Whitney *U*: *U* = 24, *p* = .163), although the lower abundance and richness of invertebrates reduced the confidence in this pattern. There was no discernable pattern in temperature affinity of fish species and their relative abundance in the different habitats (Mann–Whitney *U*: *U* = 142, *p* = .556).

**FIGURE 6:**
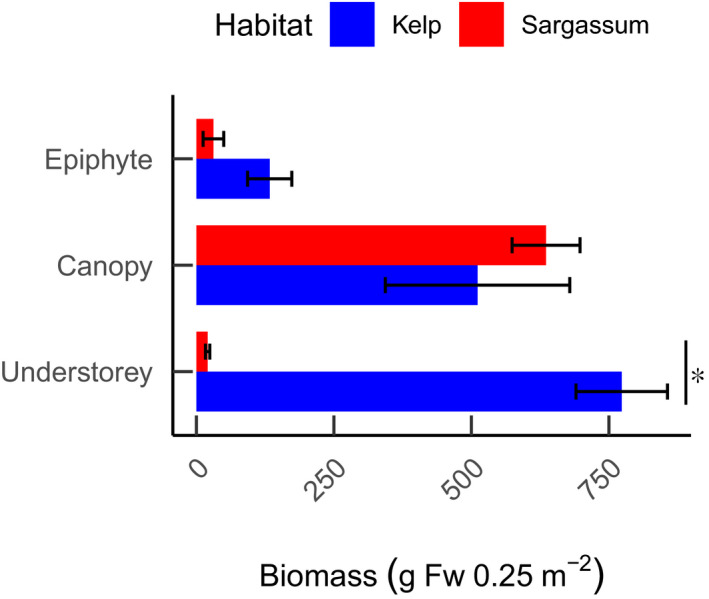
Mean seaweed biomass in different layers of the canopy in the Kelp (blue) and Sargassum (red) habitats in the Abrolhos. Error bars indicate standard error, *n* = 18 per habitat, and vertical line with asterisk indicates significant difference (GLMM, *p* < .05, see Table 1)

For both habitats, the biomass of canopy‐forming seaweeds was roughly similar (GLMM; *t* = 0.359, *p* = .720; Figure 6). However, the biomass of understory seaweeds was ~40 times higher in the Kelp habitats (GLMM; *t* = 5.986, *p* < .001) and represented the majority of the total seaweed biomass. The relative amount of biomass of epiphytes to total biomass was low in both habitats, constituting 9.4% and 4.5% of the total biomass in the Kelp and *Sargassum* habitats, respectively.

The biggest dissimilarity between habitats for the mobile invertebrate community was the abundance of the urchin *C*. *tenuispinus* (SIMPER 48.3%; Table S3). There was a low average density (0.05 ± 0.03 individuals m^−2^) of *C*. *tenuispinus* in the *Sargassum* habitats compared with that measured in the Kelp habitats (1.19 ± 0.30 individuals m^−2^). The highest density at a single site was 2.16 ± 0.65 individuals m^−2^. Corals were more abundant (GLMM; *t* = 2.112, *p* = .017) and more diverse (GLMM; *t* = 2.388, *p* < .035) in the *Sargassum* habitat. The most abundant coral found in the Kelp habitats was *Acanthastrea echinata*, which was not encountered in the *Sargassum* habitat (Figure 5).

**FIGURE 7:**
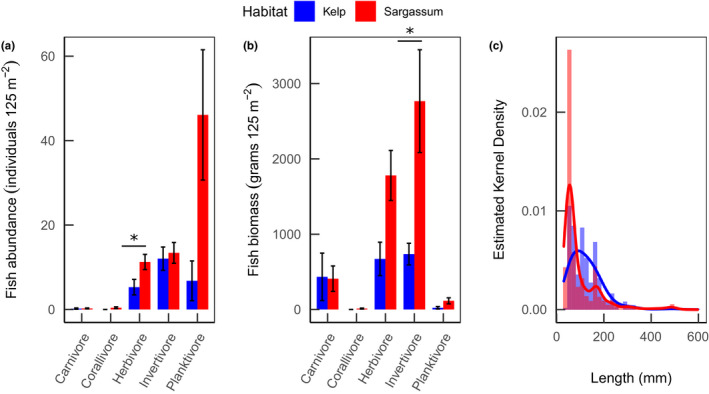
The abundance (a) and biomass (b) of different trophic guilds of fish, and kernel density of the length of fish individuals (c) in Kelp (blue) and Sargassum (red) habitats in the Abrolhos. Error bars indicate standard error, and asterisk indicates significant difference (GLMM, *p* < .05, see Table 1)

There were around two times as many herbivorous fish (Figure 7a)—accounting for double the biomass (Figure 7b)—in the *Sargassum* compared with the Kelp habitats. While the count of invertivorous fish was similar in both habitats, the individual size and biomass of invertivores was four times higher in the *Sargassum* habitats. Carnivorous fish had the highest biomass per individual, making up to ~25% and ~12.5% of the total biomass in the Kelp and *Sargassum* habitats, respectively, despite occurring in low numbers in each habitat. The planktivorous fish group showed the lowest individual biomass but occurred in high numbers. The abundance of these planktivores in the *Sargassum* habitats drove a peak in abundance of fish around 60 mm of length (Figure 7c), while in the Kelp habitats, where there were relatively few planktivores, the main size peak was for fish around 100 mm. Altogether, there was a ~40% dissimilarity between the size distribution of fish between habitats (Kolmogorov–Smirnoff test, *D* = 0.429, *p* < .001).

## 4 DISCUSSION

Here, we compared the assemblage structure of seaweed, coral, mobile invertebrate, and fish associated with habitats dominated by the kelp *Ecklonia radiata* and *Sargassum* to understand their functional redundancy and the potential effects of a shift from temperate to tropical habitat providers. The seaweed communities associated with the Kelp habitats were more temperate compared with the Sargassum habitats, as there was an overabundance of species with cool temperature affinities. The higher abundance and biomass of seaweeds in the Kelp habitat also suggests it could be more productive (Reed et al., 2008). However, the high abundance of primary producers did not correlate with the abundance of associated fauna. While mobile invertebrate abundance did not differ significantly between habitats, the abundance and biomass of fish was higher in the *Sargassum* habitats. This energy discrepancy could indicate that either there is a high turnover rate at the lower trophic levels (Stevenson et al., 2007) or secondary production is not limited by primary production within the system. It is possible that there is energy imported from nearby habitats to supplement the autochthonous energy production (Krumhansl & Scheibling, 2012; Vanderklift & Wernberg, 2008) or that fish spend time in the *Sargassum* habitats but forage elsewhere.

Aside from higher biomass, there was also a higher diversity of seaweed species in the Kelp habitat. This was further accentuated by the lack of diversity at genus level in the *Sargassum* habitats, as most species were members of the *Sargassum* genus. Low species richness can result in a low functional diversity (McWilliam et al., 2018). As congeners, the phylogenetic and morphological similarities between *Sargassum* species are expected to result in the provision of similar functions to the ecosystem. The lack of diversity would imply that there is a simplification in the *Sargassum* habitat compared with that of the Kelp, which is reflected in the lower epiphyte and understorey biomass of the seaweed habitat. Despite *Sargassum* normally hosting an abundant assemblage of epiphytes (Jacobucci et al., 2009), *Hypnea* spp. (the main epiphyte recorded) was more abundant in the heterogeneous seaweed community in the Kelp habitat. The abundant understorey seaweeds could be outcompeting corals in the Kelp habitats (Edwards & Connell, 2012), contributing to the lower coral abundance here.

We expected that high biomass of seaweeds would provide more complex habitat and result in higher abundance of associated fish (Stephens et al., 2006; Trebilco et al., 2015). However, our findings show that the lower seaweed biomass *Sargassum* habitats supported more fish, suggesting that the differences between habitats vary as a function of their interaction with the different seaweeds (Beas‐Luna et al., 2020). The prevalence of invertivorous fish in these habitats could be indicating that the fish are consuming epifauna more than the seaweeds themselves, and *Sargassum* microhabitats have been reported to harbor more mobile epifauna than kelp (Fraser et al., 2020). Furthermore, it is possible that the structural complexity could be contributing to the low number of small fish counts, as there is more shelter for smaller fish to hide in, and SCUBA divers operating stereo‐DOV systems have been found to induce avoidance behavior in fish (Watson & Harvey, 2007), with smaller fish exhibiting stronger predator avoidance behavior (Kulbicki, 1998). Alternatively, the high abundance of smaller fish could indicate that the *Sargassum* habitats are acting as nurseries for juvenile fish. Tropical seaweed habitats have been found to provide shelter for juvenile fish, which in later life stages live in other seascapes such as the kelp forests, or nearby coral reefs (Fulton et al., 2020). Regardless of the mechanism, these findings suggest both habitats differ somewhat in ecological function in terms of size and number of associated fish.

Despite these differences in invertebrate and fish abundance and body size between habitats, the invertebrate and fish species used both habitats more or less equally, regardless of their thermal affinities. This is consistent with the rapid movement and persistence of tropical herbivores in temperate ecosystems (Vergés, Steinberg, et al., 2014). Additionally, over 75% of the fish species found in the Kelp habitat were also present in the *Sargassum* habitat, suggesting that there is some functional redundancy between habitats. Indeed, a parallel is found in Californian kelp forests, where few fish species are exclusively found in Kelp habitats and not in barrens or other rocky reef habitats (Graham, 2004; Stephens et al., 2006). Alternative to the bottom‐up hypothesis of temperature controlling the seaweed distribution, the higher abundance of herbivores (*e*.*g*., parrotfish) in the *Sargassum* habitat could be controlling seaweeds top‐down. In this alternative hypothesis, temperate seaweeds—which are less resistant to herbivory—are selectively targeted by herbivores (Bolser & Hay, 1996), while more unpalatable *Sargassum* species thrive as opportunists due to the competitive release (de Eston & Bussab, 1990).

There was, however, no sign of top‐down control by invertebrates in the Kelp habitat, despite high densities of urchin *C*. *tenuispinus*. A congener of *Centrostephanus rodgersi*, which is responsible for large urchin barrens on the east coast of Australia (Andrew & Underwood, 1989; Ling & Johnson, 2009), *C*. *tenuispinus* is linked to maintaining a canopy free state on Hall Bank near Marmion, WA (Thomson & Frisch, 2010). Although the densities recorded in the Abrolhos are higher than other locations along the WA, they are still lower than those recorded on Hall Bank (~5 individuals m^−2^) (Thomson & Frisch, 2010), or on the east coast (~2–3 individuals m^−2^) where barrens persist (Andrew & Underwood, 1989; Ling & Johnson, 2009).

Despite the similarities in temperature profiles, the seaweeds in each habitat showed a discrete separation of temperature affinity, with cool‐affinity species found in the Kelp habitat. The higher variability in sea temperatures measured in the Kelp habitat suggests that there are short sporadic cooling events occurring at these sites, which could be allowing the temperate seaweeds to thrive here (Pearce, 1997). Specifically, in contrast to *Sargassum*, *E*. *radiata* is more sensitive to consistently elevated temperatures and has higher survival at variable temperatures even if higher (Straub et al., 2021). Despite the suppression of upwelling by the Leeuwin Current (Twomey et al., 2007), sporadic localized upwelling can occur under the right circumstances, and there are more upwelling days per year in the Abrolhos compared with the rest of the WA coast (Rossi et al., 2013). This local upwelling could also act as a vector for propagule dispersal from more deeper, cooler seaweed communities and bringing more diversity (Giraldo‐Ospina et al., 2021). Alternatively, the large amplitude internal waves (LAIW) may reduce the heat stress on shallow subtidal ecosystems (Reid et al., 2019). During the 2010–2011 MHW in the Eastern Indian Ocean, corals at LAIW‐exposed sites were less impacted than those on sheltered sites (Schmidt et al., 2016). The higher chlorophyll concentration in the Kelp habitat could be possible through either mechanism.

One of the main limitations of this study is the lack of temporal data. All sampling was done during a single season (Australasian Spring) and therefore does not account for phenological differences between *Sargassum* with an annual life cycle, and more perennial species such as *E*. *radiata*. While seasonal changes in, for example, biomass and life stage are relevant in *E*. *radiata* (De Bettignies et al., 2013; Wernberg & Goldberg, 2008), the relative differences between seasons in *Sargassum* can be an order of magnitude larger (Marks et al., 2018). As such, our findings do not account for expected seasonal changes or succession cycles within any of the communities (Fulton et al., 2014; Wilson et al., 2014). The low total seaweed biomass and low epiphyte biomass seen in the *Sargassum* habitat could be indicative of a young canopy, which makes the difference between habitats appear larger than it would when averaged out over a full year. Therefore, this study is strongest when viewed as a cross‐sectional community analysis of characteristic temperate versus tropical seaweed habitats in which these differences in phenology are inherent. Due to the remoteness of the investigated area, small‐scale anthropogenic influences are expected to be minimal, and the findings represent mainly the effects of the more pervasive direct and indirect effects of changes in canopy structure, for example, due to warming oceans.

We conclude that shifts from one to another foundation seaweed will impact the associated flora and fauna within these habitats. Using the presented temperate kelp forests to more tropical *Sargassum* meadows, in a space‐for‐time substitution model, indicates that increasingly warming oceans could result in a reduction of seaweed abundance and diversity, but a promotion of fish abundance and diversity at progressively higher latitudes. The discreteness found in the temperature affinity was restricted to the seaweeds and was not found in the mobile invertebrates or fish communities, suggesting tropical seaweed habitats provide a degree of functional redundancy to temperate‐affinity associated fauna. The lack of temporal data makes it difficult to discern whether the kelp forest and *Sargassum* meadows in the Abrolhos are two coexisting steady states, or whether the *Sargassum* dominance is the result of a gradual shift away from kelp forests. However, these shifts from temperate to tropical habitats are occurring along the WA coast, and are expected to continue as global warming keeps intensifying. Our results suggest that, while there will be marked changes in overall community structure, many temperate fauna could persist following a shift from kelp to *Sargassum*‐dominated habitats.

## AUTHOR CONTRIBUTION

**Yannick Mulders:** Conceptualization (lead); Data curation (lead); Formal analysis (lead); Funding acquisition (lead); Investigation (lead); Methodology (equal); Project administration (lead); Writing – original draft (lead); Writing – review & editing (supporting). **Karen Filbee‐Dexter:** Conceptualization (supporting); Data curation (supporting); Formal analysis (supporting); Methodology (equal); Writing – original draft (supporting); Writing – review & editing (lead). **Sahira Bell:** Data curation (supporting); Formal analysis (supporting); Methodology (supporting); Writing – review & editing (supporting). **Nestor E. Bosch:** Data curation (supporting); Formal analysis (supporting); Methodology (supporting); Software (supporting); Writing – review & editing (supporting). **Albert Pessarrodona:** Data curation (supporting); Methodology (supporting); Writing – review & editing (supporting). **Defne Sahin:** Data curation (supporting); Formal analysis (supporting); Methodology (supporting); Validation (supporting); Writing – review & editing (supporting). **Sofie Vranken:** Conceptualization (supporting); Data curation (supporting); Writing – review & editing (supporting). **Salvador Zarco‐Perello:** Data curation (supporting); Writing – review & editing (supporting). **Thomas Wernberg:** Conceptualization (supporting); Data curation (supporting); Investigation (supporting); Methodology (equal); Supervision (lead); Validation (lead); Writing – review & editing (lead).

## Supporting information

Table S1‐S5

## Data Availability

All satellite data are available from IMOS through the AODN portal: https://portal.aodn.org.au/.
