# Supplementary material for: Intergrading reef communities across discrete seaweed habitats in a temperate–tropical transition zone: Lessons for species reshuffling in a warming ocean
Source: Ecol Evol. 2022 Jan 24;12(1):e8538. doi: 10.1002/ece3.8538 (PMC8796930; doi:10.1002/ece3.8538)
Supplement: Supplementary file 1 — Table S1‐S5 [file ECE3-12-e8538-s001.docx]

Table S1: Two way factorial ANOVA output comparing the monthly mean sea surface temperature (SST), minimum SST, difference between mean and minimum SST, and chlorophyll concentration between Kelp and Sargassum habitats at the Abrolhos from 1992 to 2019. Habitat (2 levels) and month (12 levels) were fixed effects, and monthly value each year was a random effect.

| **Model** | **Sum Sq** | **Df** | **F value** | **Pr(>F)** |
| --- | --- | --- | --- | --- |
| *SST* |  |  |  |  |
| Habitat | 2.46 | 1 | 4.956 | **0.026** |
| Month | 1118.91 | 11 | 204.870 | **< 0.001** |
| Habitat x Month | 3.59 | 11 | 0.657 | 0.779 |
| Residuals | 317.27 | 639 |  |  |
| *Minimum SST* |  |  |  |  |
| Habitat | 0.03 | 1 | 0.034 | 0.854 |
| Month | 993.20 | 11 | 107.248 | **< 0.001** |
| Habitat x Month | 8.57 | 11 | 0.925 | 0.515 |
| Residuals | 537.97 | 639 |  |  |
| *Delta min-mean SST* |  |  |  |  |
| Habitat | 3.02 | 1 | 8.932 | **0.003** |
| Month | 6.48 | 11 | 1.743 | 0.061 |
| Habitat x Month | 2.91 | 11 | 0.783 | 0.657 |
| Residuals | 216.07 | 639 |  |  |
| *Chlorophyll* |  |  |  |  |
| Habitat | 1.37 | 1 | 47.099 | **< 0.001** |
| Month | 5.51 | 11 | 17.280 | **< 0.001** |
| Habitat x Month | 0.99 | 11 | 3.115 | **< 0.001** |
| Residuals | 8.73 | 301 |  |  |

Table S2: Species specific contribution to the Bray-Curtis dissimilarities of seaweed biomass (log transformed) between Kelp and Sargassum habitats at the Abrolhos, as well as the thermal affinity and layer in the canopy for each species.

| **Species** | **Average** | **Std. dev** | **Ratio** | **Kelp**  **Habitat** | **Sargassum**  **Habitat** | **Cumulative dissimilarity %** | **Affinity** | **Layer** |
| --- | --- | --- | --- | --- | --- | --- | --- | --- |
| *Sargassum linearifolium* | 0.079 | 0.027 | 2.915 | 0.00 | 5.17 | 8.7 | Widespread | Canopy |
| *Pterocladia lucida* | 0.059 | 0.027 | 2.187 | 4.01 | 0.00 | 15.1 | Cool | Understorey |
| *Sargassum ligulatum* | 0.047 | 0.035 | 1.364 | 0.08 | 3.07 | 20.3 | Warm | Canopy |
| *Hennedya crispa* | 0.047 | 0.026 | 1.772 | 3.19 | 0.00 | 25.4 | Cool | Understorey |
| *Plocamium sp.* | 0.046 | 0.031 | 1.519 | 3.01 | 0.01 | 30.5 | Cool | Understorey |
| *Callophycus oppositifolius* | 0.046 | 0.038 | 1.220 | 3.28 | 0.00 | 35.6 | Cool | Understorey |
| *Sargassum fallax* | 0.046 | 0.040 | 1.141 | 2.82 | 2.41 | 40.6 | Cool | Canopy |
| *Hypnea spp.* | 0.044 | 0.029 | 1.531 | 3.47 | 1.18 | 45.5 | Widespread | Epiphyte |
| *Ecklonia radiata* | 0.044 | 0.049 | 0.893 | 2.74 | 0.00 | 50.3 | Cool | Canopy |
| *Delisea pulchra* | 0.031 | 0.034 | 0.894 | 2.11 | 0.00 | 53.6 | Cool | Understorey |
| *Lobophora variegata* | 0.028 | 0.014 | 1.966 | 0.51 | 2.23 | 56.8 | Warm | Understorey |
| *Zonaria spiralis* | 0.025 | 0.029 | 0.848 | 1.61 | 0.00 | 59.5 | Cool | Understorey |
| *Euptilota articulata* | 0.024 | 0.024 | 0.996 | 1.67 | 0.00 | 62.1 | Cool | Understorey |
| *Haloplegma sp.* | 0.022 | 0.016 | 1.379 | 1.49 | 0.00 | 64.5 | Widespread | Understorey |
| *Sargassum ilicifolium* | 0.020 | 0.032 | 0.639 | 0.00 | 1.34 | 66.8 | Warm | Canopy |
| *Betaphycus speciosum* | 0.020 | 0.039 | 0.502 | 1.11 | 0.13 | 68.9 | Warm | Understorey |
| *Heterodoxia denticulata* | 0.018 | 0.022 | 0.836 | 1.34 | 0.00 | 71.0 | Cool | Understorey |
| *Glossophora nigricans* | 0.017 | 0.022 | 0.787 | 1.29 | 0.00 | 72.9 | Cool | Understorey |
| *Dictyota sp.* | 0.016 | 0.014 | 1.205 | 1.15 | 0.53 | 74.7 | Widespread | Understorey |

Table S3: Species specific contribution to the Bray-Curtis dissimilarities of Invertebrate abundance (log transformed) between Kelp and Sargassum habitats at the Abrolhos, as well as the thermal affinity for each species.

| **Species** | **Average** | **Std. dev** | **Ratio** | **Kelp**  **Habitat** | **Sargassum**  **Habitat** | **Cumulative dissimilarity %** | **Affinity** |
| --- | --- | --- | --- | --- | --- | --- | --- |
| *Centrostephanus tenuispinus* | 0.430 | 0.252 | 1.707 | 5.93 | 0.27 | 48.3 | Cool |
| *Tectus* | 0.103 | 0.140 | 0.739 | 0.40 | 0.93 | 59.9 | Warm |
| *Angaria tyria* | 0.103 | 0.130 | 0.790 | 0.20 | 1.07 | 71.5 | Warm |
| *Crinoidae* | 0.100 | 0.121 | 0.827 | 0.40 | 0.93 | 82.7 | Widespread |
| *Latirus turritus* | 0.042 | 0.084 | 0.499 | 0.00 | 0.40 | 87.4 | Warm |
| *Chromodoris westrauliensis* | 0.025 | 0.044 | 0.562 | 0.13 | 0.20 | 90.2 | Cool |
| *Conidae* | 0.014 | 0.041 | 0.353 | 0.00 | 0.20 | 91.8 | Widespread |
| *Fromia indica* | 0.014 | 0.040 | 0.355 | 0.00 | 0.13 | 93.4 | Warm |
| *Astralium* | 0.012 | 0.034 | 0.360 | 0.00 | 0.13 | 94.8 | Cool |
| *Pentanogaster dubeni* | 0.010 | 0.025 | 0.386 | 0.20 | 0.00 | 95.9 | Cool |
| *Muricidae* | 0.009 | 0.040 | 0.236 | 0.00 | 0.07 | 97.0 | Widespread |
| *Turbo jourdani* | 0.009 | 0.034 | 0.254 | 0.07 | 0.00 | 97.9 | Cool |

Table S4: Species specific contribution to the Bray-Curtis dissimilarities of fish abundance (log transformed) between Kelp and Sargassum habitats at the Abrolhos, as well as the thermal affinity and Diet group for each species.

| **Species** | **Average** | **Std. dev** | **Ratio** | **Kelp**  **Habitat** | **Sargassum**  **Habitat** | **Cumulative dissimilarity %** | **Affinity** | **Diet** |
| --- | --- | --- | --- | --- | --- | --- | --- | --- |
| *Scarus schlegeli* | 0.094 | 0.072 | 1.309 | 0.62 | 1.70 | 12.5 | Cool | Herbivore |
| *Chromis westaustralis* | 0.089 | 0.100 | 0.887 | 0.54 | 1.73 | 24.3 | Warm | Planktivore |
| *Coris auricularis* | 0.080 | 0.080 | 1.001 | 1.21 | 1.57 | 35.0 | Cool | Invertivore |
| *Anampses geographicus* | 0.061 | 0.057 | 1.070 | 1.05 | 0.56 | 43.1 | Widespread | Invertivore |
| *Parma occidentalis* | 0.039 | 0.044 | 0.894 | 0.49 | 0.58 | 48.3 | Widespread | Herbivore |
| *Scarus ghobban* | 0.039 | 0.037 | 1.056 | 0.15 | 0.77 | 53.5 | Warm | Herbivore |
| *Choerodon rubescens* | 0.031 | 0.034 | 0.898 | 0.30 | 0.50 | 57.6 | Widespread | Herbivore |
| *Thalassoma lutescens* | 0.025 | 0.035 | 0.704 | 0.38 | 0.19 | 60.9 | Warm | Invertivore |
| *Notolabrus parilus* | 0.024 | 0.034 | 0.700 | 0.24 | 0.31 | 64.1 | Cool | Invertivore |
| *Parupeneus spilurus* | 0.021 | 0.030 | 0.689 | 0.14 | 0.39 | 66.8 | Cool | Invertivore |
| *Thalassoma lunare* | 0.020 | 0.030 | 0.654 | 0.22 | 0.25 | 69.5 | Warm | Invertivore |
| *Austrolabrus maculatus* | 0.020 | 0.032 | 0.614 | 0.23 | 0.17 | 72.1 | Cool | Invertivore |
| *Hemigymnus fasciatus* | 0.018 | 0.033 | 0.529 | 0.25 | 0.06 | 74.4 | Warm | Invertivore |
| *Plectorhinchus flavomaculatus* | 0.017 | 0.031 | 0.559 | 0.06 | 0.25 | 76.7 | Warm | Invertivore |
| *Parma mccullochi* | 0.016 | 0.026 | 0.630 | 0.09 | 0.30 | 78.8 | Cool | Invertivore |
| *Plectropomus leopardus* | 0.014 | 0.033 | 0.414 | 0.10 | 0.16 | 80.7 | Warm | Herbivore |
| *Chaetodon plebeius* | 0.011 | 0.027 | 0.416 | 0.00 | 0.20 | 82.1 | Warm | Carnivore |
| *Plectorhinchus caeruleonothus* | 0.010 | 0.025 | 0.392 | 0.00 | 0.13 | 83.4 | Warm | Corallivore |
| *Halichoeres brownfieldi* | 0.010 | 0.026 | 0.364 | 0.13 | 0.00 | 84.7 | Cool | Invertivore |
| *Lethrinus nebulosus* | 0.010 | 0.020 | 0.476 | 0.00 | 0.23 | 86.0 | Warm | Invertivore |

Table S5: Species specific contribution to the Bray-Curtis dissimilarities of coral abundance between Kelp and Sargassum habitats at the Abrolhos, as well as the thermal affinity for each species.

| **Species** | **Average** | **Std. dev** | **Ratio** | **Kelp Habitat** | **Sargassum Habitat** | **Cumulative dissimilarity %** | **Affinity** |
| --- | --- | --- | --- | --- | --- | --- | --- |
| *Favites sp.* | 0.297 | 0.261 | 1.137 | 0.00 | 1.83 | 31.6 | Widespread |
| *Montipora grisea* | 0.197 | 0.188 | 1.043 | 0.67 | 0.58 | 52.5 | Warm |
| *Turbinaria sp.* | 0.164 | 0.249 | 0.660 | 0.00 | 1.00 | 70.0 | Widespread |
| *Cyphastrea serailia* | 0.077 | 0.114 | 0.678 | 0.33 | 0.25 | 78.2 | Warm |
| *Acanthastrea echinata* | 0.066 | 0.113 | 0.589 | 0.33 | 0.00 | 85.3 | Warm |
| *Paragoniastrea australensis* | 0.051 | 0.081 | 0.634 | 0.00 | 0.58 | 90.7 | Widespread |
| *Platygyra deadalea* | 0.037 | 0.067 | 0.553 | 0.00 | 0.25 | 94.7 | Warm |
| *Favia sp.* | 0.025 | 0.059 | 0.422 | 0.00 | 0.25 | 97.3 | Widespread |
| *Astreopora myriophthalma* | 0.015 | 0.035 | 0.429 | 0.00 | 0.25 | 98.9 | Warm |
| *Moseleya latistellata* | 0.006 | 0.020 | 0.297 | 0.00 | 0.08 | 99.5 | Warm |
| *Acropora hyacinthus* | 0.005 | 0.015 | 0.297 | 0.00 | 0.08 | 100.0 | Warm |
